# Can Old Animals Reveal New Targets? The Aging and Degenerating Brain as a New Precision Medicine Opportunity for Epilepsy

**DOI:** 10.3389/fneur.2022.833624

**Published:** 2022-04-28

**Authors:** Aaron del Pozo, Leanne Lehmann, Kevin M. Knox, Melissa Barker-Haliski

**Affiliations:** Department of Pharmacy, School of Pharmacy, University of Washington, Seattle, WA, United States

**Keywords:** late-onset epilepsy, animal models, neuroinflammation, Alzheimer's disease, stroke

## Abstract

Older people represent the fastest growing group with epilepsy diagnosis. For example, cerebrovascular disease may underlie roughly 30–50% of epilepsy in older adults and seizures are also an underrecognized comorbidity of Alzheimer's disease (AD). As a result, up to 10% of nursing home residents may take antiseizure medicines (ASMs). Despite the greater incidence of epilepsy in older individuals and increased risk of comorbid seizures in people with AD, aged animals with seizures are strikingly underrepresented in epilepsy drug discovery practice. Increased integration of aged animals into preclinical epilepsy drug discovery could better inform the potential tolerability and pharmacokinetic interactions in aged individuals as the global population becomes increasingly older. Quite simply, the ASMs on the market today were brought forth based on efficacy in young adult, neurologically intact rodents; preclinical information concerning the efficacy and safety of promising ASMs is not routinely evaluated in aged animals. Integrating aged animals more often into basic epilepsy research may also uncover novel treatments for hyperexcitability. For example, cannabidiol and fenfluramine demonstrated clear efficacy in syndrome-specific pediatric models that led to a paradigm shift in the perceived value of pediatric models for ASM discovery practice; aged rodents with seizures or rodents with aging-related neuropathology represent an untapped resource that could similarly change epilepsy drug discovery. This review, therefore, summarizes how aged rodent models have thus far been used for epilepsy research, what studies have been conducted to assess ASM efficacy in aged rodent seizure and epilepsy models, and lastly to identify remaining gaps to engage aging-related neurological disease models for ASM discovery, which may simultaneously reveal novel mechanisms associated with epilepsy.

## Epilepsy and Seizures

Seizures and epilepsy have a high incidence worldwide. At least 10% of the population will experience seizures at some point in life (1). Many factors can contribute to a seizure: metabolic disorders (2), alcohol withdrawal (3), illicit drug use (4), acute neurological insults, or high fever (1, 5). Regardless of the inciting mechanism, seizures can be generalized or focal (6, 7). On the other hand, epilepsy is a chronic disease with a cumulative incidence of 3% worldwide. Many factors increase the risk of developing epilepsy: congenital disorders (5), infections (8), trauma (9), tumors (10), vascular accidents (11), and neurodegenerative diseases (12, 13). The incidence of focal seizures increases significantly in individuals aged 65+ (14), resulting in a higher socioeconomic impact for this group vs. younger individuals (15). Epilepsy in older adults can arise secondary to other disorders; ~10–20% of cases of epilepsy syndromes are comorbid with neurodegenerative diseases, such as Alzheimer's Disease (AD) (13, 16). Despite this, the epileptic syndromes most frequently studied in preclinical practice generally favor disease or symptoms that occur in infant or adolescent stages of neurodevelopment, (17, 18). However, despite the fact that older populations have an equal or higher prevalence of epilepsy compared with younger populations (16), comprehensive studies in older animals are rather limited (19, 20). Altogether, epilepsy in older adults is a problem that is more frequently documented in clinical settings than is investigated in pre-clinical practice.

## Drug-Resistant Epilepsy: the Importance of Aging

Preclinical antiseizure drug discovery is booming (21, 22). Numerous antiseizure medicines (ASMs) improve the quality of life in people with epilepsy by reducing the number of seizures, as well as generally increasing seizure threshold (19, 23–25). ASMs can regulate the expression and activity of ion channels (especially sodium and calcium channels), inhibit hyperexcitability by increasing GABA levels and/or reducing glutamate levels or regulate the trafficking and release of other neurotransmitters (26). However, about one-third of people with epilepsy suffer from uncontrolled seizures despite pharmacotherapy and are considered “drug-resistant” (27, 28); i.e., when the control of seizures is not achieved with at least two or three appropriately chosen ASMs. One of the most severe consequences of uncontrolled chronic seizures is the increased risk for sudden unexpected death in epilepsy (SUDEP). Unfortunately, the precise mechanisms of drug-resistant epilepsy (DRE) are still unknown. Despite the approval of many new ASMs in the last 20+ years, the percentage of people with DRE has not significantly changed (29). New strategies are needed to improve twenty-first century ASM discovery and meaningfully shift this percentage.

Seizure vulnerability is not uniform with advanced age (15, 16), therefore pharmacological considerations must also be addressed for this group. Older populations respond more effectively to pharmacological treatment than younger populations (30–32). The higher efficacy in seizure control among older people with epilepsy may be due, in part, to age-related differences in etiology. For example, perampanel administration to older people with epilepsy leads to a 50% responder rate of 57% (33), whereas this responder rate is 35% in younger people. At the same time, the absence of ASM treatment may exacerbate the burden of neurodegenerative diseases that have comorbid seizures, such as AD (13, 34). However, older people with epilepsy are at considerable risk for drug-drug interactions due to changes in renal, hepatic, or metabolic function. There must be, as a result, careful consideration of the ASMs to be used for older people with epilepsy. Aged seizure and epilepsy pre-clinical models could be appropriate to elucidate the differences between the old and young brain, determine the potential for adverse effects liability, and identify the genes and pathways involved in both epilepsy and neurodegenerative disorders. Precision medicine approaches for epilepsy to-date have relied on the identification of novel therapies in syndrome-specific models of genetically acquired pediatric epilepsy, with a high degree of success translating those discoveries to clinical populations [e.g., fenfluramine (35) and cannabidiol (36, 37)]. Whether older people with epilepsy represent a similarly rich population to model diverse causes of hyperexcitability and potentially uncover novel pharmacological targets for disease represents an untapped opportunity.

Unfortunately, most studies on epilepsy have employed younger adult animals; the study of epilepsy (and other comorbid conditions) in aged animals is rather limited (32, 38). Thus, this review will update the studies on epilepsy and seizures performed in aged rodents, describe the existing gaps in the study of epilepsy, and propose new targets relevant to the aged brain, information which may help to increase the understanding of epilepsy and discover new pharmacological therapies that are useful in a diversity of epilepsy syndromes.

## The Relationship Between Aging and Epilepsy

Modeling the sequelae of epilepsy in aged animals is challenging from a variety of perspectives, including species-specific physiological changes with aging, the interaction of comorbidities observed in people with epilepsy, and the feasibility and cost limitations of inducing clinically relevant neurological disease in aged animals. These factors limit current models, especially concerning the replication of age-related conditions (32, 38).

### Studies of Seizures and Epilepsy in Aged Mice

Most of the studies conducted in older animals have compared seizure susceptibility between sexes (Table 1). Few have explored the relationship between aging and epilepsy. Table 1 details the studies that have been conducted since 2010 (32) and their major findings summarized.

**Table 1:** Primary pre-clinical studies of epilepsy in aged mice.

**Mouse strain**	**Seizure induction model**	**Age (months) and sex (M/F)**	**Primary scientific objective**	**Principle study conclusions**
C57BL/6	Kainic acid	4 and 20 (M)	Evaluate the differences in susceptibility to KA stimulus between old and young mice	Aged animals have more astrogliosis and enhanced susceptibility to developing epilepsy vs. young animals (39)
C57BL/6J & FVB/NF	Kainic acid	2, 12, and 18 (M)	Evaluate how the susceptibility to developing seizures changes over time	Susceptibility to kainic acid-induced epilepsy increased during the time of study (40)
C57BL/6 TAU-	Pentylenetetrazol	24 (M&F)	Identify the role of Tau on epilepsy and seizures in aged animals	Inhibition of Tau exerts a neuroprotective effect by reducing the number of seizures in aged rodents (41)
fyn^−/−^/tau^−/−^ (C57BL/6J)	Pentylenetetrazol	3 (M&F)	Identify the effects of genetic deletion of Fyn and/or tau on seizure severity	Tau and fyn have an important impact on early epileptogenesis (42)
C57BL/6	Hippocampal (CA3) kindling	3 and 18–22 (M)	Evaluate the effect of aging on sensitivity to hippocampal kindling	Sensitivity to CA3 hippocampal kindling increases with advanced age (43)
C57BL/6	Hippocampal kindling	12–14 (M)	Understand the mechanisms of TLE on the aged hippocampus	Extended kindling of the hippocampus in old mice is a valid model of TLE (44)
PLCG1F/F (C57BL/6)	Phospholipase Cγ-1 delection	10–16 (M/F)	Identify the role of PLCγ 1 in epilepsy	Genetic deletion of PLCγ1 leads to handling-induced seizures in aged mice (45)
C57BL/6	Hypoxic ischemic insult	18–20 (M)	Evaluate the anticonvulsant effect of lorazepam and fosphenytoin in hypoxia- ischemia insult in aged animals	HI insult produces epilepsy, and the drug treatment is only effective if administered before the insult (46)
C57BL/6 + STROKE	Stroke insult	16–20 (M)	Study the mechanisms of epilepsy secondary to stroke	Stroke induces epilepsy, and the treatment is not effective (47)
C57BL/6 RHEB-	mTOR pathway	Up to 24 (M&F)	Identify the role of the mTOR pathway in epilepsy	Interfering with the mTOR pathway can induce seizures in elderly animals (48)
C57BL/6	Natural, age-related changes in hippocampal excitability	1, 16–18, and 24–28 (–)	Examine age-dependent changes in hippocampal network activity	Hyperexcitability in the hippocampus increases with age (49)

*M, male; F, female; -, indicates sex not specified*.

#### Pentylenetetrazol (PTZ) Studies

The chemoconvulsant pentylenetetrazol (PTZ), has provided insight into some of the pathways and genes involved in epilepsy in the aged and degenerating brain (41). In the PTZ model, older mice required a lower dose of this chemoconvulsant vs. younger mice; older mice responded to GABA_A_ agonists better than juvenile mice, mimicking patient responses (50). Further, PTZ-induced epilepsy in accelerated senescence mice (SAM) affects different cell populations, brain areas, and neurotransmitters relative to younger animals (51). During the juvenile and adult stage, the primary cell population affected by PTZ administration is astrocytes, whereas in aged animals, this cell population are neurons. Notably, the GABAergic pathway to prevent seizures in older animals was found to be particularly relevant to prevent seizures (51).

The relevance of tau in aging-related epilepsy has also been interrogated using the acute PTZ seizure model. Tau, a microtubule-associated protein, is a natively unfolded protein in human brains and has several biological roles, including microtubule assembly and stabilization (52). There are age-accelerated changes in neurofibrillary tangle (hyperphosphorylated tau) pathology in people with chronic DRE compared to age-matched individuals without epilepsy (53, 54). Aged tau-knockout (KO) mice that received PTZ administration have increased latency for acute PTZ-induced seizures and reduced seizure severity compared to controls (41). Putra and colleagues confirmed these findings with aged tau KO mice, but then went on to demonstrate that simultaneous deletion of Fyn, a member of the Src family tyrosine kinases (SFKs) that interacts with tau protein, also beneficially alters PTZ-induced seizures severity and latency (42). This beneficial effect of tau deletion seems to be due to the relationship of tau over hyperexcitability, neuroinflammation, and neuronal death (41, 42). These studies reveal that this AD-associated protein and tau-related pathophysiological mechanisms are a relevant target in epilepsy. Thus, PTZ-induced seizures evoked in old mice may affect different cell populations than in younger mice.

#### KA Studies

Kainic acid (KA)-induced status epilepticus (SE) in mice revealed that some of the most affected areas secondary to seizure induction are the hippocampus, cortex, amygdala, and thalamus (39, 40). Nonetheless, vulnerability to seizures is higher in aged mice, supporting the bimodal distribution of seizure susceptibility in the very young and the very old (40). Furthermore, in FVB mice, seizure stimulation results in greater cell death with advanced age vs. younger animals. Further, glia may play an important role in setting the epileptogenic threshold and determining levels of neurodegeneration (39, 40). In FVB/NJ SAM (32, 55, 56) or melatonin III KO mice (57), the electroconvulsive shock (58) or KA administration increases brain damage (specifically in the hippocampus), as well as the susceptibility to epilepsy in aged animals (39, 40, 58). While studies have more recently also assessed the contributions of tau in the intra-amygdala KA-induced SE model evoked in young-adult mice (59), similar studies to assess the additive impacts of KA-SE and AD-associated proteins (i.e., tau) have yet to be reported in aged animals. Thus, the KA SE model is useful to assess the consequences of aging on seizure susceptibility and epilepsy, as well as to understand the pathophysiological pathways and mechanisms involved.

#### Kindling Studies

Kindling of aged mice elicits similar biological outcomes as the chemoconvulsants mentioned above. The severity of and susceptibility to hippocampal and CA3 kindling in old C57BL/6 mice increases with advanced age (43, 44). Moreover, these animals not only present alterations on the EEG but also develop cognitive impairment (43, 44). Thus, hippocampal degeneration may explain the detrimental effect of epilepsy in older people with AD (60, 61). Post-mortem confirmed AD cases also exhibit reduced pyramidal cell counts in the hippocampus (62); whether chronic seizures in an aging model evoke similar hippocampal deficits is worthy of further scrutiny.

#### Other Studies

Aligning with clinical risk factors for epilepsy in old individuals (16), traumatic brain injury (TBI) and stroke are risk factors for epilepsy in aged animals (46, 47). In animal models, stroke-induced epilepsy can also result in death. Thus, animals with stroke or hypoxic-ischemic insult may not only be a valid model to study the effect of age on epilepsy in mouse models (Table 1), but they may also represent a possible SUDEP model (46, 47).

The Ras homolog enriched in brain protein (RHEB; one of the major proteins involved in the mTOR pathway; Table 1) may represent a novel driver of age- and sex-dependent susceptibility to handling-induced seizures (48). This mutation is mechanistically related to everolimus, which is approved as a disease-modifying treatment for tuberous sclerosis complex, a condition characterized by chronic seizures and epilepsy in pediatric populations. Rapalogues, such as everolimus, have been found to be disease-modifying in some (63), but not all (25, 64, 65), animal models of acquired spontaneous recurrent seizures. Notably, the mTOR pathway also plays a critical role in epilepsy and seizures susceptibility in aged rodents (48), but further investigations are certainly needed to more comprehensively define the role of mTOR signaling in spontaneous seizures in aged mice.

Phospholipase Cγ-1 protein in mice (a protein expressed in GABAergic neurons) may explain some differences in epilepsy development between young and older individuals (45). Aged mice with selective deletion of phospholipase Cγ-1 in GABAergic neurons have increased susceptibility to handling-induced spontaneous seizures (45). Further, aged mice with selective phospholipase Cγ-1 deletion in GABAergic neurons have increased spontaneous seizures, but reduced anxiety and deficits in contextual fear memory, despite the reduction in inhibitory synapses observed in the hippocampus (45). Thus, the GABAergic pathway may be particularly relevant to spontaneous seizures, but not epilepsy-related behavioral comorbidities, in aged animals.

Other studies revealed that there is a progressive age-related increase in hippocampal hyperexcitability (specifically in CA3). Variation in the levels of the proto-oncogene c-Fos, responsible, among other things, for synaptic plasticity (49) and the modification of glial phenotype and function are some of the causes that may be involved in this progressive increase in hyperexcitability. Moreover, age-related changes in gliosis and synaptic plasticity can impact the integrity of synapses and neurons (66).

In conclusion, although few additional aged mouse studies have been reported since the last comprehensive review was published (32), these studies reported have shown that the release of neurotransmitters, synaptic plasticity, and metabolic pathways enhance seizure susceptibility with advanced age (Table 1). Despite the differences in mice strain, response to epileptic stimuli, and experimental seizure induction paradigm, these studies are nonetheless influential to broaden understanding of the pathophysiological mechanisms of seizures and epilepsy in the aged brain. These works establish new models to improve the knowledge of the mechanisms of epilepsy and its relationship with old age.

### Studies of Seizures and Epilepsy in Aged Rats

During the last decade, studies of epilepsy and aging effects in rats have been even less frequently conducted than in mice, likely as a result of advances in genetic models of aging-related neurological disorders favoring mice, as well as the financial constraints and resource limitations associated with aged rats. Nonetheless, the most notable studies carried out in recent years are summarized in Table 2.

**Table 2:** Primary pre-clinical studies of epilepsy in aged rats.

**Rat strain**	**Seizure induction model**	**Age (months) and sex (M/F)**	**Primary scientific objective**	**Principle study conclusions**
Sprague-Dawley	Pilocarpine	22 (–)	Investigate how status epilepticus in aged rats affects astrocytes in the hippocampus	Astrocyte phenotype, but not density, changes with aging (67)
Wistar	Pilocarpine	3 and 17–22 (M)	Study the effect of epilepsy on neurogenesis in young and old animals	As aging occurs, neurogenesis can still be induced in aged rats with a history of epilepsy, though in general, this neurogenesis is less than that which is seen in younger rats (68)
Fischer	Kainic acid	6–8 and 22–28 (M)	Evaluate any possible changes induced by epilepsy in the hippocampal CA3 region	Old animals have more damage in CA3 and are more vulnerable to develop seizures and epilepsy (69)
Sprague-Dawley	Kainic acid	Aged from 2 to 24 (M)	Evaluate how the brain changes from epilepsy at different ages	Similar to younger animals, aged animals with epilepsy also exhibit features of hippocampal hyperexcitability (70)
Fischer	Stroke	4 and 20 (M)	Evaluate if stroke results in epilepsy and seizures 2 months after the insult	Both young and old animals with stroke develop seizures post-insult. The volume of the ischemic lesion was independent of the number and severity of seizures (71)
F344	Kainic acid	5 and 22 (M)	Compare the neurological issues between young and aged rats secondary to KA induction	Old age in rats results in a greater loss of hippocampal CA1 pyramidal neurons, an increased propensity for developing robust chronic TLE, and severe cognitive dysfunction (72)
F344	Kainic acid	4 and 22 (–)	Compare the survival of subpopulations of hippocampal GABA-ergic interneurons	The loss of neurons is increased in older animals (73)

*M, male; F, female; -, indicates sex not specified*.

Studies in rats highlight the relevance of other pathological mechanisms that are different from those observed in mice. Pilocarpine-induced epilepsy in aged rats triggers an important switch in glial phenotype and response (67). Further, epilepsy-induced in old rats leads to a reduction of their neurogenic properties, resulting in a lower density of basal dendrites and dentate granule cells compared with younger animals (68). Moreover, KA-induced epilepsy in aged rats results in a greater loss of pyramidal neurons in CA3 compared with younger rats (72, 73). Altogether, seizures promote a massive glutamate release, oxidative stress, and inflammation, similar to the impacts of other aging-related disorders, such as AD or stroke (32, 69). Further, in parallel with mice, hippocampal hyperexcitability in aged rats is the most affected biological outcome (69, 70). Epilepsy in aged mice and rats clearly reproduce similar pathophysiology as in humans.

Clearly, there is untapped value in integrating aged models more frequently to better understand the molecular pathways of epilepsy and seizure. As we have been able to successfully uncover many novel therapeutic targets based on findings in pediatric genetic epilepsy models, it is entirely plausible that integrating aged rodents may similarly carry broad relevance to other epilepsy indications, including developmental epileptic encephalopathies (DEE).

## Potential Neurobiological Mechanisms

**Figure 1:**
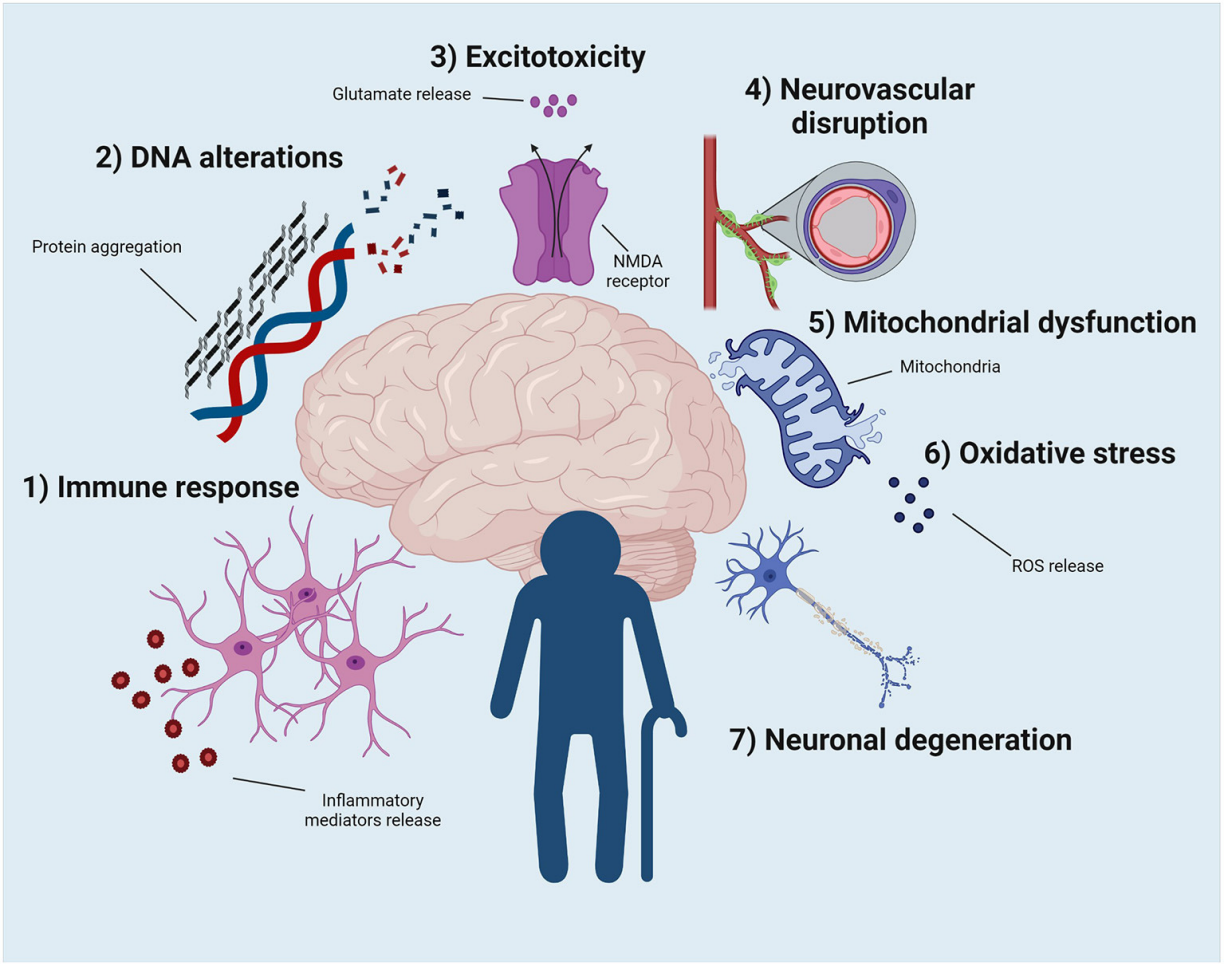
Potential physiological events that can contribute to the development of epilepsy and to the better pharmacological response of older people with epilepsy. (1) The exacerbated immune response, as well as the change in the phenotype and properties of microglia and astrocytes, seem to contribute to this phenomenon. (2) DNA modification due to protein aggregation contributes to enhance epilepsy vulnerability in the elderly population. (3) The uncontrolled release of glutamate ultimately leads to increased excitotoxicity, resulting in a new pathway that explains old brain function. (4) Blood-brain barrier permeability increases with aging. This event may increase the vulnerability to developing epilepsy but may also improve pharmacological sensitivity in older adults. (5) Mitochondrial dysfunction and oxidative stress (6) can be related to epilepsy, age, and drug response. (7) Neuronal death secondary to aging increases the susceptibility to epilepsy. Illustration created with BioRender.com under a paid subscription.

Aging, in general, is associated with cellular, molecular, anatomical, and physiological changes (74). There are likely also intrinsic processes unique to the aged brain that may promote seizure susceptibility (75). For example, age-related increases in tau and β-amyloid protein accumulation may contribute to the enhanced incidence of seizures (75), as well as glial response and DNA alterations (76, 77). Below, we will highlight the major physiological mechanisms related to brain aging and explain how these factors may bidirectionally influence susceptibility to seizures and epilepsy (Figure 1). Moreover, these biomolecular changes may identify the pathways that contribute to the better drug response seen in older people with epilepsy, as well as the potential factors that may promote DRE.

### Immune Response

Aging shifts the responsiveness of the immune system (16, 78), i.e., immunosenescence. With aging, there is decreased immune competence to respond to a stimulus and a generally elevated inflammatory state (78, 79). Although the biological mechanisms involved are the subject of active investigation and beyond the scope of this review, aging affects the phenotype and function of the cells comprising innate and adaptive immune cells (78). In both humans and rodents of advanced age, inflammation increases in different areas of the brain (16, 78, 80–82) (Figure 1). In aged individuals, microglia and astrocytes promote inflammatory cytokine release; glial cells also have reduced phagocytic properties (16, 78, 80–82). Additionally, the release of anti-inflammatory cytokines in aged individuals is significantly lower than in younger populations. As in other neurological disorders, the immune response is not only restricted to the brain in epilepsy. Neutrophils and monocytes/macrophages in older adults show impaired phagocytosis, chemotaxis, and immune capacity in response to injury or pathogenic signal in the periphery (16, 78, 80–82). Studying the interaction between the brain and systemic inflammation in older people is thus essential to reproduce the course of different pathologies, such as epilepsy. It is worth mentioning that this effect is sex-dependent, such that estradiol helps to improve the function of immune system cells until menopause occurs, wherein the aging of the immune environment begins (78).

Microglia, particularly when activated, are the primary source of inflammatory cytokines in the brain. IL-1, IL-6, and IFN-α levels are increased in the brains of old vs. younger adult mice and rats (78, 83). Additionally, primed and activated microglia residing in the aged brain often show an exacerbated response to external stimuli, such as infection or stress (78, 83). Peripheral markers of inflammation, including serum cytokine levels, are also elevated in older population. In addition, peripheral inflammation serves as a risk factor for the development of age-related neurodegenerative disease (78, 83) and may play a primary role in the etiology or progression of age-associated pathologies. Thus, inflammation may play an important role in aging-related disorders, as it does in as epilepsy and seizures.

### Protein Dysregulation

Proteins are repaired by the chaperone system and eliminated by autophagy or ubiquitin systems (78, 84). These systems become compromised with advanced age. Folding and accumulation of aberrant protein aggregates are thus common in aged individuals (78, 84) (Figure 1). Protein folding or aggregation can have a detrimental effect on the cell, resulting in loss or gain of function. Indeed, β-amyloid and tau proteins progressively aggregate with age, and are associated with the presence of cellular dysfunction, degeneration, and epilepsy (78, 84).

The aggregation of tau and β-amyloid, two proteins implicated in AD, can similarly explain the close relationship between neurodegeneration and epilepsy. Although the mechanisms by which these proteins may act on epilepsy are not fully understood, tau reduces PP2A activity, increasing GSK3β and CDK5 activity, activating PI3K/AKT pathways, and promotes NMDAR subtypes to drive glutamate release (85). These findings support the role of Aβ and tau in epilepsy and indicate that these neurodegenerative proteins may be relevant therapeutic targets in older people with epilepsy.

### Oxidative Stress

Aging and oxidative stress are closely related (78) (Figure 1). Aging is the progressive loss of tissue and organ function over time (86). The free radical or the oxidative stress theory of aging, is based on the structural damage-based hypothesis that age-associated functional losses are due to the accumulation of oxidative damage to macromolecules (i.e., lipids, DNA, and proteins) by reactive oxygen species (ROS) (86). The exact mechanism of oxidative stress-induced aging is still not clear but is likely due to increased ROS levels leading to cellular senescence, a physiological mechanism that stops cellular proliferation in response to damages that occur during replication. Senescent cells promote the secretion of soluble factors (interleukins, chemokines, and growth factors), degradative enzymes and insoluble proteins/extracellular matrix components (86).

Studies of naturally aged rodents and genetic SAM models demonstrate that there is an accumulation of oxidation products in the brain over time (78). Oxidative stress is the result of the aggregation of ROS (87, 88). Several factors are involved in ROS accumulation (78). However, a drastic reduction in the levels of antioxidant enzymes, such as superoxide dismutase, and increased inflammation particularly impact epilepsy in the aged brain (87, 88).

SE changes the redox potential and decreases ATP levels, reducing the production and supply of energy to the brain (89). Moreover, epilepsy reduces antioxidant enzyme levels (NADH or FADH) and increases ROS production (89). Indeed, nitric oxide synthase expression is increased in the early phases of epileptogenesis in mice (90). Acute, seizure-induced reductions in hydrogen sulfide levels are detected in young adult mice during the development of the corneal kindled model of epilepsy, and similar studies in aged mice are needed (90). Thus, oxidative stress is highly associated with both aging and epilepsy.

### Mitochondrial Dysfunction

Mitochondrial dysfunction is another pathological feature of both aging and epilepsy (78, 91) (Figure 1), which can lead to endogenous production of ROS and apoptosis (74). Mitochondria fulfill their diverse functions partly through constant remodeling via fission and fusion (78, 91). Dysregulation of mitochondrial fission and fusion contributes to numerous types of nervous system disease, including epilepsy (78, 91) Scavenging of ROS may be a valid anticonvulsant strategy (92), in general. Mitochondrial quality control is compromised with aging, and there is evidence of reduced mitochondrial membrane potential, probably due to alteration of the DNA encoding the essential subunits of the electron transport chain. All these detrimental factors increase ROS accumulation (78, 91). Mitochondrial mRNA and protein expression are dynamically altered in response to epileptogenesis in the young-adult brain (90), but whether this is similarly impacted by age remains to be determined. Unfortunately, few studies have sufficiently assessed how aging and epilepsy additively or synergistically influence mitochondrial membrane integrity dynamics or quality control.

### Excitotoxicity

Excitotoxicity describes the toxic actions of excitatory neurotransmitters, mainly glutamate, where exacerbated or prolonged activation of glutamate receptors initiates a cascade of neurotoxicity that ultimately leads to loss of neuronal function and cell death (88, 93) (Figure 1). Both inflammation and oxidative stress can induce massive glutamate release. During aging, the brain is in an environment in which both inflammation and oxidative stress play a key role, producing, among other things, excitotoxic damage (88). Neurodegenerative changes associated with human epilepsy, may in part, result from persistent glutamatergic activity (94). The repetitive depolarization of glutamate terminals causes release of excess glutamate resulting in excitotoxic degeneration of the postsynaptic neuron (94). Astrocytes, known to be responsible for the maintenance of synaptic glutamate levels, display phenotype and function changes with advanced age (95). As a result, excitotoxicity and late-onset epilepsy may be closely interrelated.

### Neurovascular Dysfunction

The blood-brain barrier (BBB) consists of endothelial cells, tight junctions, astrocytes, pericytes, and the extracellular matrix (78, 96–98). Together, these elements maintain cerebral permeability to blood components and protect the brain from infiltration of neurotoxic compounds (78, 96–98). Physiological aging is associated with deterioration of the cerebrovascular system and increased BBB permeability, making the brain more vulnerable to neurological insults (78). Similarly, aging-related oxidative stress, inflammation, and excitotoxicity contribute to increased BBB permeability (97) (Figure 1).

Although BBB disruption is associated with other neurological disorders, increased permeability may be relevant in understanding differences in drug response between older and younger people with epilepsy. The increased permeability may contribute to better drug uptake, resulting in differential response (99). This effect has already been observed in the treatment of brain tumors. Treatment with BBB disruptors in people with brain tumors promotes a better response to traditional drugs, improving disease prognosis (99). For this reason, the CNS bioavailability in people with epilepsy may be insufficient and may be one mechanism underlying DRE (28). Age-related differences in BBB permeability could contribute to differences in drug response in old vs. young people with epilepsy, but rigorous preclinical studies to assess this are needed.

### DNA Alterations and Cell Death

Aging is accompanied by progressive atrophy of tissues and aggregation of stochastic damage to DNA, RNA, protein, and lipid macromolecules (78, 100). Loss of genomic maintenance may contribute causally to aging (Figure 1). Several premature aging syndromes have underlying genetic defects in DNA repair (78, 100). Accumulation of DNA damage may be particularly prevalent in the CNS due to low DNA repair capacity in post-mitotic brain tissue (78, 100). In general, the cumulative effects of detrimental changes that occur in aging, primarily after the reproductive phase, are thought to contribute to species-specific aging rates. In addition, there is also abundant evidence of a causal link between mitochondrial DNA damage and aging phenotypes (78, 100).

In epilepsy, studies have not yet conclusively revealed the effect of an acute seizure on neuronal death and the role of seizure-induced neuronal death in acquired epileptogenesis. Isolated seizures probably do not kill neurons. However, severe, and repetitive seizures do [i.e., SE (78, 100)]. Acquired epileptogenesis does not necessarily require neuronal death (78, 100). Models with chronic seizures (e.g., kindling) are not always defined by neurodegeneration (101) in the early phases of disease, but are nonetheless valid epilepsy models that are incredibly useful to evaluate disease mechanisms and seizure susceptibility (20, 102–104), comorbidities (105), and identify therapeutic agents (106). Indeed, the absence of neuronal death is difficult, if not impossible, to prove, so studies are increasingly challenging the concept that loss of synaptic input from dying neurons is critical for inducing axonal sprouting and synaptic circuit reorganization (78, 100). An alternative hypothesis may be more relevant; biochemical pathways causing programmed neurodegeneration, rather than neuronal death, are responsible for, or contribute to, epileptogenesis. The reprogramming of neuronal death pathways derives from necroptosis or pyroptosis (a form of cell death triggered by inflammatory signals and associated with inflammation) (78, 100). This hypothesis explains why neuronal death appears to be closely related to epileptogenesis, but not always (107). Age-related neurodegeneration likely increases seizure susceptibility, but more studies will better define how these two pathological processes functionally converge.

### Neurotrophic and Neurotransmitter Contributions to Aging

Normal aging evokes molecular and cellular changes in the brain that may also delay recovery after injury (74). Aging decreases monoamine densities due to alterations in neuronal number, synapses, and protein levels. Indeed, the aging brain has altered the expression of neurotrophic factors and neurotransmitters: i.e., brain-derived neurotrophic factor, norepinephrine, and serotonin (74, 108). Lastly, elevated monoamine oxidase is associated with the release of free radicals that can also further promote neuronal death via apoptosis (74).

Preclinical and clinical evidence increasingly supports that serotonin (5-HT) has an important role in epilepsy and SUDEP (109, 110). Clinical studies have focused on the effect of 5-HT agonism on seizure semiology, frequency, and autonomic function, whereas studies in animal models have focused on the effect of restoring 5-HT signaling on seizure susceptibility and/or seizure-associated mortality (111). Increased 5-HT levels may generally protect against seizures and SUDEP. Conversely, uncontrolled seizures and epilepsy may reduce 5-HT tone and elevate risk of SUDEP (111–113). The clinical efficacy of 5-HT reuptake inhibitors, such as fluoxetine or citalopram, to control seizures or prevent SUDEP is still controversial (104, 114).

Epilepsy in older people is particularly concerning when it arises due to comorbid conditions because of an elevated mortality risk. Mortality specifically from AD in people with epilepsy has increased over 216% in the last 20 years, whereas people with epilepsy alone have seen a significant (27%) decrease in mortality (115). Whether epilepsy and AD are similarly impacted by changes in 5-HT-related signaling, which is known to be associated with SUDEP risk, remains to be determined.

Aging causes substantial biological changes that play an important role in other related neurological disorders, such as epilepsy or AD. These physiological events may be relevant to understand differential sensitivity to ASMs of older adults with epilepsy compared to younger individuals, as well as elucidate the reason for the increased incidence of epilepsy in this demographic.

## Relationship Between Epilepsy and Aging-Related Neurological Disorders

Older adults represent the fastest-growing demographic with epilepsy (16, 34). While seizures in older people with epilepsy are generally not resistant to available ASMs, many pathological processes can contribute to hyperexcitability. In this regard, aged rodent models are an important resource to better understand mechanisms of epilepsy and identify novel and broadly relevant therapeutic treatments.

New strategies for innovative epilepsy drug discovery and/or prevention are needed. A disruptive strategy to ASM discovery came from the US FDA-approval of cannabidiol in 2018. While early limited clinical studies had suggested potential anticonvulsant efficacy (116), there were few well-controlled studies in human subjects or animal models. The seminal demonstration of efficacy of this agent came from a pediatric epileptic syndrome-specific model, the *Scn1a*^+/−^ mouse model of Dravet syndrome (36). This supported cannabidiol's further characterization in established seizure and epilepsy models in wild-type, adult rodents (37) that had otherwise advanced the clinical study of *all* FDA-approved ASMs on the market until that point (19). Similarly, fenfluramine was initially identified in a zebrafish model of Dravet syndrome (35) and subsequently approved by the US FDA in 2020. As a result, syndrome-specific pediatric models will assume increased prominence in twenty-first century ASM discovery. These pediatric models present an unparalleled opportunity to identify novel molecular targets and contributors to epilepsy that may be broadly relevant to epileptogenesis and ictogenesis (117). However, we herein argue that this approach could be similarly applied to the opposite end of life. Translational science must better align the use of preclinical models with the heterogeneity of clinical epilepsy (118). In the twentieth century, numerous models of acute seizure in seizure naïve and wild-type rodents were indispensable to the identification of symptomatic treatments. In the twenty-first century, impactful therapies for epilepsy will be discovered in disease models that represent all facets by which epilepsy can arise.

To address this untapped opportunity, additional emphasis on aging-related models of epilepsy affords a novel precision medicine strategy for early drug discovery and/or to identify new molecular contributors to epilepsy, in general. Many of the animal models used to understand the effect of epilepsy in the context of aging involve pathways that are also relevant to the pathophysiology of cerebrovascular disease (i.e., stroke) or AD (13, 32). A high percentage of patients usually develop epilepsy and seizures because of either condition (16). Age exacerbates the associated pathological factors. We propose a strategy of increased prioritization of aged animals in epilepsy research and drug discovery, with an explicit focus on the two primary drivers of epilepsy in older adults: cerebrovascular disease and AD.

### Stroke

Stroke and cerebrovascular disease are highly associated with epilepsy (16, 71, 119). Post-stroke seizure refers to seizures or epilepsy that develops after a stroke without a previous history of epilepsy, which is due to reversible or irreversible damage caused by the ischemic or hemorrhagic infarction and not to other structural brain abnormalities or metabolic disorders (119). Stroke is the most identified cause of epilepsy in adults older than 35 years. In older adults, stroke accounts for more than half of the newly diagnosed cases of epilepsy in which a cause is determined, ahead of degenerative disorders, brain tumors, and head trauma (120). From stroke registry data, about 5–20% of all individuals who have a stroke will have subsequent seizures, but epilepsy (recurrent seizures) will develop in only a small subset of this group (120). Each year, more than 730,000 people in the United States have a stroke; a yearly conservative incidence of post-stroke seizures is about 36,500 new cases per year (120).

### Alzheimer's Disease

Epilepsy is an under-recognized comorbidity of AD (13). Roughly 44 million people worldwide live with AD. Of those, around 5% have early-onset AD (EOAD), which arises due to a mutation in one of three deterministic risk genes: amyloid precursor protein (APP), Presenilin-1 (PSEN1), or Presenilin-2 (PSEN2). The precise mechanisms leading to the development of seizures in AD are still under investigation and have been extensively reviewed elsewhere (13, 121). Nonetheless, the relative risk of unprovoked seizures is markedly increased in people with EOAD-associated risk factors, reaching up to 87 times the risk of seizures in individuals with AD onset between the ages of 50 and 59 years vs. the general population. For comparison, the relative incidence of Dravet syndrome is 1:15,700 individuals in the U.S, whereas early-onset AD incidence is estimated to be 13.2 per 100,000/year in individuals aged 30–64 (1:7,575 individuals) (122), highlighting a significant disparity in the allocation of resources to investigate a major contributor to epilepsy, as well as an untapped opportunity to identify new molecular targets that could be broadly relevant to a variety of epilepsy syndromes.

## Recommendations for Incorporating Aged Animal Models of Epilepsy

Unfortunately, the number of preclinical studies of the effect of aging on epilepsy is limited. There are several plausible limitations. One reason is the resource limitations associated with mimicking the physiological conditions of aging itself. Second, aging naturally deteriorates biological resilience and processes. The coexistence of chronic comorbidities, such as high cholesterol or hypertension, results in a “new physiological state” that is difficult to mimic in rodents. Further, such reduced systems as rodent models may infrequently consider additive impacts such as environmental conditions to reduce variables in a reasonably powered study. Third, older adults (over 65 years of age) are a more frequently polymedicated population (123, 124). Identifying the potential therapeutic effect of an investigational agent in an aging-related disorder model with the added consideration of polypharmacy is an experimental constraint. The cost of keeping older animals is more expensive compared with younger animals. Finally, studying older animals simply takes a lot of time. Investigators must anticipate experiments more in advance than if they were performed in young-adult animal models. These factors altogether limit the feasibility of old preclinical models, reducing capacity to identify new potential drugs or targets.

Ultimately, twenty-first century epilepsy drug discovery could strategically rely on developing new animal models based on the mutations or pathophysiological events produced in both stroke and AD to better reproduce late-onset epilepsy in an efficient manner. These models would fill the above-detailed preclinical void. Interdisciplinary integration of clinical observations into preclinical research would dynamically improve translational research (118). Rather than a reliance on a bench-to-patient approach that has defined twentieth century ASM discovery, twenty-first century efforts must rely on clinical findings to inform translational scientific investigation for epilepsy. Advanced age negatively influences epilepsy susceptibility. Thus, the study of epilepsy in aged animal models remains an area of considerable opportunity for future therapeutic innovation.

We propose that some of the characteristics that animal models should have to study the role of epilepsy in advanced age and vice versa are:

- Use a range of ages of “old” animals to understand heterogeneity in the geriatric population. This includes rodents older than 16 months.
- Studies should be performed with both sexes to dissect the age-related effects of gonadal hormones or other sex-specific factors on functional outcomes and susceptibility to epilepsy.
- Use epilepsy models that phenotypically reproduce the symptomatology observed in older people with epilepsy, including those age-associated comorbidities such as stroke and AD-associated genotypes.
- Increase emphasis on establishing a pharmacokinetic/pharmacodynamic relationship in aged animals to inform on the potential for adverse effects liability in clinical populations. This is particularly critical to older population, who have decreased renal and hepatic function and may be subject to polypharmacy.
- Study the response to repeated seizures both acutely and chronically.
- Include biomarkers to validate of age-related changes that are translationally feasible.

## Conclusions

Older adults are more vulnerable to seizures and epilepsy, but less resistant to ASMs than young people with epilepsy. Preclinical studies in aged animals and the effect of advanced age on epilepsy and seizures are unfortunately limited. Little work has been conducted to define ASM efficacy in preclinical models of late-onset epilepsy. This review updates the comprehensive review by Kelly (32) and extends upon that report to highlight new avenues for therapeutic innovation. We suggest incorporation of aged animals and new pathways and pathophysiological mechanisms involved in epilepsy and seizures in the aged and degenerating brain, which may vary with the age of the individuals. If nothing else, the intensity and effort of the epilepsy research community should match the clinical need in this patient group to a similar degree as has, in recent years, been dedicated to pediatric patients, whose seizure incidence is similar (13). Likewise, this review highlights the importance of acting on the biomolecular mechanisms that naturally vary with advanced age because they play a fundamental role in age-related neurological disorders. Finally, we conclude that, as in humans, animals with stroke or AD variants could be more frequently integrated into the study of mechanisms of late-onset epilepsy, which may extend more broadly to other syndromes and advance new therapeutic targets to people with epilepsy.

## Author Contributions

AP, LL, KK, and MB-H contributed to the literature review and writing of this manuscript. All authors contributed to the article and approved the submitted version.

## Funding

This work was supported by ITHS KL2 (KL2TR002317) and R01AG067788 to MB-H.

## Conflict of Interest

The authors declare that the research was conducted in the absence of any commercial or financial relationships that could be construed as a potential conflict of interest.

## Publisher's Note

All claims expressed in this article are solely those of the authors and do not necessarily represent those of their affiliated organizations, or those of the publisher, the editors and the reviewers. Any product that may be evaluated in this article, or claim that may be made by its manufacturer, is not guaranteed or endorsed by the publisher.
